# Putting a Hold on the Downward Spiral of Paranoia in the Social World: A Randomized Controlled Trial of Mindfulness-Based Cognitive Therapy in Individuals with a History of Depression

**DOI:** 10.1371/journal.pone.0066747

**Published:** 2013-06-27

**Authors:** Dina Collip, Nicole Geschwind, Frenk Peeters, Inez Myin-Germeys, Jim van Os, Marieke Wichers

**Affiliations:** 1 Department of Psychiatry and Psychology, School of Mental Health and Neuroscience, Maastricht University Medical Centre, Maastricht, The Netherlands; 2 Health Psychology Group, CLEP, University of Leuven, Leuven, Belgium; 3 Department of Psychosis Studies, Institute of Psychiatry, King's Health Partners, King's College London, London, United Kingdom; Chiba University Graduate School of Medicine, Japan

## Abstract

**Context:**

Paranoia embodies altered representation of the social environment, fuelling altered feelings of social acceptance leading to further mistrust. Mindfulness-based cognitive therapy (MBCT) may relieve paranoia and reduce its impact on social acceptance.

**Objective:**

To determine whether MBCT alters momentary feeling of paranoia and social acceptance in daily life.

**Design:**

Randomized controlled trial of daily-life repeated measures (up to 120 per participant) before and after allocation to MBCT or waiting list control.

**Participants:**

Volunteer sample of 130 eligible men and women with residual affective dysregulation after at least one episode of major depressive disorder.

**Interventions:**

Eight weeks of MBCT in groups of 10–15 participants in addition to participants' usual treatment.

**Outcome Measures:**

Daily-life ratings of paranoia and social acceptance. This manuscript concerns additional analyses of the original trial; hypotheses were developed after data collection (focus initially on depressive symptoms) but before data analysis.

**Results:**

Sixty-six participants were assigned to the waiting list control group and 64 to the MBCT intervention group, of whom 66 and 61 respectively were included in the per-protocol analyses. Intention-to-treat analyses revealed a significant group by time interaction in the model of momentary paranoia (b = −.18, p<0.001, d = −0.35) and social acceptance (b = .26, p<0.001, d = 0.41). Paranoia levels in the intervention group were significantly reduced (b = −.11, p<0.001) and feelings of social acceptance significantly increased (b = .18, p<0.001), whereas in the Control condition a significant increase in paranoia (b = .07, p = 0.008) and a decrease in social acceptance was apparent (b = −.09, p = 0.013). The detrimental effect of paranoia on social acceptance was significantly reduced in the MBCT, but not the control group (group by time interaction: b = .12, p = 0.022).

**Conclusions:**

MBCT confers a substantial benefit on subclinical paranoia and may interrupt the *social* processes that maintain and foster paranoia in individuals with residual affective dysregulation.

**Trial Registration:**

Netherlands Trial Register NTR1084

## Introduction

Humans have always been social animals. The social world is an important source of satisfaction and support for most of us. However, social contact can also be a source of suffering and problems. Individuals with a diagnosis of mental disorder often have difficulties to adapt to the social world and to trust others [1]. Although representations of social mistrust are common also in the general population, they generally take on more severe forms in the context of mental disorders [2]. Two important factors which fuel paranoia are low self-esteem [3], expectation of mistreatment and affective dysregulation [4], [5], illustrating the transdiagnostic nature of paranoia. Paranoia consequently is not only commonly observed in individuals with psychosis but also in individuals with affective disorders such as depression, [5], [6]. The presence of paranoid symptoms impacts negatively on outcome even when expressed at subclinical level [7], and increases the risk for developing a full-blown psychotic disorder [8]. Paranoia affects representations of the social environment, resulting in unease in social situations and reduced social approach. The resulting social avoidance will further fuel mistrust, giving rise to a downward spiral [9]. Recent research proposes that mindfulness-based approaches, for instance Mindfulness-Based Cognitive Therapy (MBCT) and Acceptance and Commitment Therapy (ACT), are effective in treating different forms of psychopathology. Most studies were conducted in patients with depression or anxiety disorder [10], [11], but a few small randomized controlled trials have reported positive effects of ACT and MBCT for patients recovering from acute psychosis [12], [13], [14], [15], [16], [17], [18]. There is little work on possible psychological processes that might be transformed by of mindfulness-based therapies [19], [20], particularly in terms of examining alterations in the subtle dynamic interplay of altered representations and social interaction in real life, for example when people interact with others, or when they feel suspicious. Real-time and real-world assessment approaches such as the Experience Sampling Method (ESM) have been optimized for the investigation of dynamic patterns of interaction between experience and behaviour [21].

Mindfulness-based approaches, unlike cognitive therapy, are not aimed at changing the contents of mental experience, but rather target the relationship the individual has with thoughts and feelings [22]. Participants practice to become more aware of moment-to-moment sensations and to recognize negative feelings/thoughts, without being *wrapped up* in them or considering them to be a reflection of reality. This practice may bring about the ability to deal with feelings of insecurity and paranoia.

In a sample of participants with affective dysregulation and increased risk to experience feelings of mistrust, ESM was used to measure feelings of paranoia and appraisal of social interactions during six days before and six days after either MBCT or waiting list control group (hereafter: Control). This study used data pertaining to a published RCT on the effects of MBCT on depressive symptoms [23], to test further hypotheses, developed after completion of the RCT, regarding the effects of MBCT on paranoia. The primary aim of the study was to examine the effects of MBCT on feelings of paranoia and on the appraisal of social situations, measured as they occur in daily life. A secondary aim was to investigate whether MBCT may interrupt the downward spiral between feeling paranoid and feeling less accepted in social situations, using time-lagged analyses.

The hypothesis was that, following MBCT, individuals would feel (i) less paranoid and (ii) more at ease during social interaction. Furthermore, following MBCT, we expected (iii) a reduction of the association between paranoia and unease during social interactions.

## Methods

### Participant Characteristics

In the MindMaastricht study (trial number NTR1084, Netherlands Trial Register Trial registration: http://www.trialregister.nl/trialreg/admin/rctview.asp?TC=1084) [23], adults with residual depressive symptomatology (> = 7 on the 17-item Hamilton Depression Rating Scale; HDRS [24]) after at least one episode of Major Depressive Disorder (MDD) and thus at risk of also displaying elevated paranoid ideation [7], were recruited from outpatient mental health care facilities in Maastricht and through posters in public spaces. As the focus was on stable residual symptomatology in the context of a previous affective disorder, individuals were excluded if they met SCID criteria for a current depressive episode, schizophrenia, or any psychotic disorder in the past year, and recent (past four weeks) or upcoming changes in ongoing psychological or pharmacological treatment. Sample sociodemographic and clinical characteristics are displayed in Table 1.

**Table 1:** Baseline demographic and clinical characteristics in each group.

	MBCT (n = 63)	Control (n = 66)
Age (mean, SD)	44.6 (9.7)	43.2 (9.5)
Female gender	79%	73%
Full-/part-time work	62%	68%
Illness/unemployment benefits	19%	23%
Living with partner/own family	64%	64%
Comorbid anxiety disorder (present)	35%	49%
Comorbid anxiety disorder (past)	51%	64%
Current psycho-counseling/-therapy	13%	12%
Current use of antidepressants	32%	38%
(Occasional) use of benzodiazepines	8%	8%

*Note.* There were no significant differences between groups (at *p*<0.05). MBCT = Mindfulness-based cognitive therapy; Control = waiting list control condition.

### Sampling procedures

All study procedures were approved by the Medical Ethics Committee of Maastricht University Medical Centre, and all participants signed an informed consent form. The protocol for this trial and supporting CONSORT checklist are available as supporting information; see Checklist S1 and Protocol S1. An initial screening was performed by phone to check for availability during the study period and likelihood of meeting inclusion and exclusion criteria. A second screening included administration of the Structured Clinical Interview for DSM IV axis I (SCID-I) [25] and the 17-item HDRS by trained psychologists. Eligible participants were invited for a detailed explanation of the experience sampling procedure, and then took part in the baseline assessment. The baseline assessment consisted of six days of experience sampling in the participant's natural environment (see below). After the baseline assessment, participants were randomized to either MBCT or waiting list (allocation ratio 1∶1). After either eight weeks of MBCT (see below) or equivalent waiting time (Control condition), participants again took part in six days of experience sampling. All participants were compensated with gift vouchers worth 50 Euros. Participants in the Control condition had the opportunity to take part in MBCT after the post-intervention assessment.

### Randomization

Randomization to treatment condition was stratified according to number of depressive episodes (two or less versus three or more), as at the time of randomization studies suggested a greater benefit for those with three or more previous episodes [26]. An independent researcher not involved in the project generated the randomization sequence in blocks of five (using the sequence generator on www.random.org), and wrote the randomization code into sealed numbered envelopes. After completion of all baseline assessments, the researcher allocated participants to their treatment condition based on the randomization code in the sealed envelope (opened in order of sequence). No masking of treatment condition took place.

### Intervention (MBCT)

Content of MBCT training sessions followed the protocol of Segal, Williams, and Teasdale [27]. Training consisted of eight weekly meetings lasting 2.5 hours and were run in groups of 10–15 participants. Assessment periods for control participants were matched to those of MBCT participants. Sessions included guided meditation, experiential exercises, and discussions. In addition to the weekly group sessions, participants received CDs with guided exercises and were assigned daily homework exercises (30 to 60 minutes daily). Training sessions were given by experienced trainers in a centre specialized in mindfulness trainings. All trainers were supervised by an experienced health care professional who had trained with Teasdale and Williams, the co-developers of MBCT [28].

### Experience Sampling Method and Measures

ESM is a momentary assessment method to repeatedly assess participants in their daily living environment. Compared to retrospective questionnaires and interviews, ESM offers several advantages: (a) enhanced ecological validity, (b) minimized retrospective bias, because participants' experiences are assessed as they occur, and (c) enhanced reliability, because participants' experiences are assessed repeatedly [29]. In the current study, participants received a digital wristwatch and a set of ESM self-assessment forms collated in a booklet for each day. The wristwatch was programmed to emit a signal (“beep”) at an unpredictable moment in each of ten 90-minute time blocks between 7:30 am and 10:30 pm, on six consecutive days, resulting in a maximum of 60 beeps per study period. After each beep, participants were asked to fill out ESM self-assessment forms, collecting reports of current emotions, cognition and context. All self-assessments were rated on 7-point Likert scales (ranging from *not at all* to *very*). Trained research assistants explained the ESM procedure to the participants during an initial briefing session, and a practice form was completed to confirm that participants understood the 7-point Likert scale. Participants were instructed to complete their reports immediately after the beep, thus minimizing memory distortion, and to record the time at which they completed the form. All reports not filled in within 15 min after the actual beep were excluded from the analysis, since previous work has shown that reports completed after this interval are less reliable and consequently less valid. For the same reason, participants with less than 20 valid reports at baseline were excluded from the analysis [30]. Previous studies have demonstrated the feasibility, validity, and reliability of ESM in general and patient populations [21].

#### Momentary paranoid feelings

In accordance with previous work [31], momentary paranoia was measured with the ESM item *I feel suspicious*.

#### Momentary social acceptance

In case participants were not alone at the time of the beep, they evaluated their social company in terms of social acceptance *(In this company, I feel accepted)* [32].

### Statistical Methods

ESM data have a hierarchical structure. Thus, multiple observations (level 1) are clustered within participants (level 2). Note that assessment period (pre/post) is not a separate level but an independent factor, just as in regular regression analyses. Multilevel analyses take the variability associated with each level of nesting into account [33]. Multilevel linear regression analyses, using the XTREG command on a Windows platform in Stata 11.1 were applied to the ESM data.

First, we examined the effects of MBCT on change in paranoia and feelings of social acceptance relative to the Control condition. Thus, in the models of paranoia and feelings of social acceptance, the two-way interaction between study period (baseline vs. post assessment) and treatment group (Control vs. MBCT) was the parameter of interest.

Second, we examined whether MBCT changed the time-lagged association between paranoia and feelings of social acceptance in the flow of daily life compared to the Control condition. To this end, we analysed the three-way interaction between study period (baseline vs. post assessment), treatment group (Control vs. MBCT), and paranoia at time point t-1 on feeling accepted at time point t and respectively with feeling accepted at time point t-1 on paranoia at time point t. In case of significant interactions, stratified effects were calculated for the four different conditions (MBCT and Control, at baseline and post-intervention). Within each treatment group, statistical significance of differences between baseline and post-assessment was assessed by Wald test [34].

The reported analyses are based on the whole sample (intention to treat). Participants who attended less than four MBCT sessions (*n* = 3) were excluded for the per-protocol analysis (outcomes were similar and are not reported).

## Results

### Participants

Recruitment of participants started in January 2008 and ended in February 2009, and all post-intervention assessments were completed by August 2009, when the pre-determined number of participants was reached. Sociodemographic and clinical characteristics of MBCT and Control participants are displayed in Table 1. At baseline, there were no large or significant differences between treatment groups with respect to sociodemographic and clinical characteristics. Table 2 shows baseline and post assessment scores of variables used in the analyses, stratified by treatment group. Again, there were no large or significant differences between groups at baseline. The baseline overall mean of momentary paranoia was 1.65 (SD = 1.27, range = 1–7). Participant flow through the study can be found in an earlier publication [35]. No known harms or unintended treatment effects were reported in either group.

**Table 2:** Baseline and post-assessment means and standard deviations of experience sampling variables used in the analyses, stratified by group.

	MBCT (n = 63)						CONTROL (n = 66)					
	baseline		post		change p-value	Cohen's d	baseline		post		change p-value	Cohen's d
	M	SD	M	SD			M	SD	M	SD		
I feel suspicious	1.63	1.27	1.48	1.04	<0.001	−.13	1.67	1.28	1.72	1.25	0.008	.04
I feel accepted	5.91	1.19	6.09	1.06	<0.001	.16	5.98	1.28	5.88	1.2	0.013	−.08
MBCT sessions attended			7.2	1.5								
Minutes practiced per day			29.7	13.2								

Note. MBCT = Mindfulness-based cognitive therapy plus treatment as usual; Control = waiting list control condition (treatment as usual); M = mean; SD = Standard deviation.

Participants completed 12,453 ESM entries. Of these, 559 (4%) were excluded as invalid entries, because completion times fell outside the pre-determined window of 15 minutes after the beep. On average, participants completed 49 (of 60; *SD* = 7.6) valid entries per ESM assessment period. One participant had completed fewer than 20 valid entries at baseline and was therefore excluded from the analyses.

### Effects of MBCT on paranoia and social acceptance

Multilevel analysis revealed a significant interaction between treatment group and study period in the model of momentary paranoia (b = −.18; 95%CI −.25, −.10, p<0.001; d = −0.35); the MBCT group reported significantly reduced feelings of paranoia from pre- to post-assessment (b = −.11; 95%CI −.16, −.05, p<0.001), whereas in the Control condition a significant increase was apparent (b = .07; 95%CI .02, .12, p = 0.008).

Similarly, significant interactions between treatment group and study period were apparent for the variable *I feel accepted* (b = .26; 95%CI .16, .36, p<0.001; d = 0.41). Thus, MBCT participants felt more accepted after the MBCT training (b = .18; 95%CI .13, .25, p<0.001), compared to before, whereas Control participants felt less accepted after the waiting period (b = −.09; 95%CI −.16, .02, p = 0.013). For baseline and post-assessment means and effect sizes, see Table 2.

### MBCT and time-lagged associations between paranoia and feeling accepted

**Figure 1:**
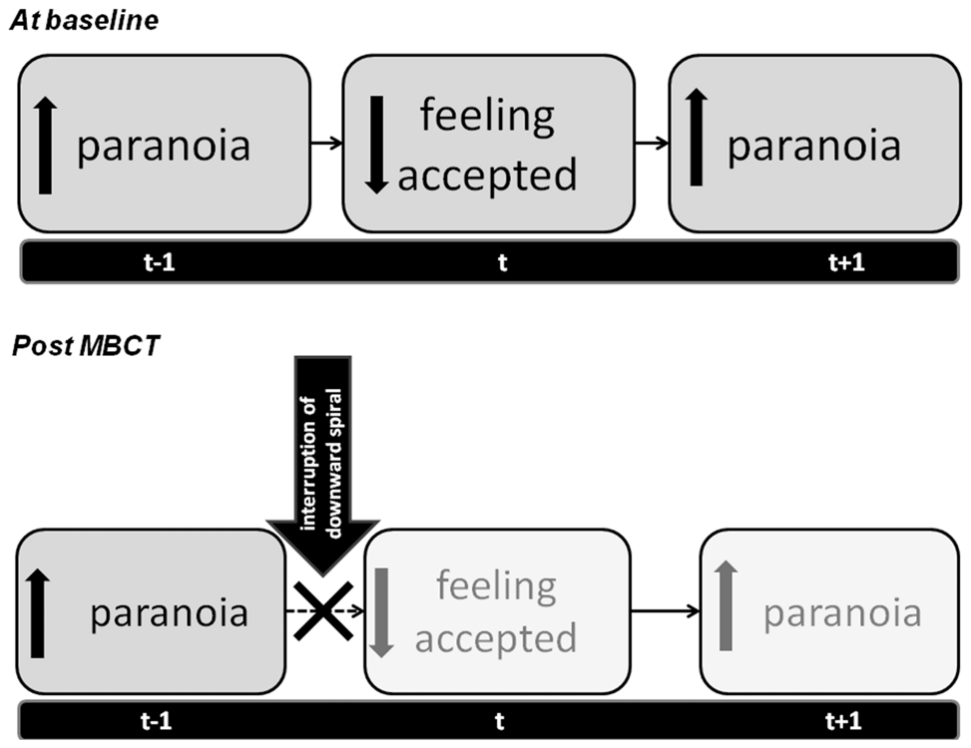
Time-lagged associations between feeling paranoid and feeling accepted in the flow of daily life: baseline and post-Mindfulness-based cognitive therapy (MBCT).

**Figure 2:**
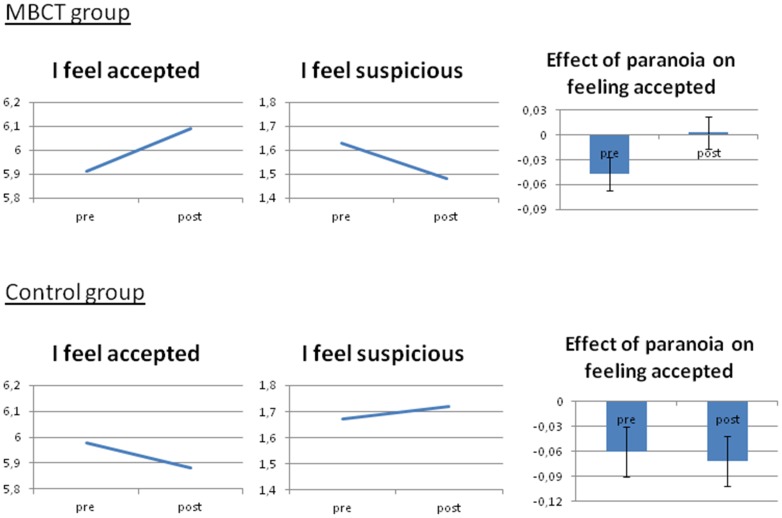
The effect of Mindfulness-based cognitive therapy (MBCT) on feeling accepted, feeling paranoid and their association compared to the control condition: Overview of the main results.

MBCT compared to Control was associated with a significant change in the effect of paranoia on prospective feelings of social acceptance between pre- and post-treatment (b = .12; 95%CI .02, .21, p = 0.022). Before MBCT, feeling more suspicious was associated with feeling less accepted the next moment (b = −.06; 95%CI −.11, −.02, p = 0.002). After MBCT, the association between feeling suspicious at t-1 and feeling less accepted at t disappeared (b = .03; 95%CI −.03, .10, p = 0.30)(Figure 1 and Figure 2), while in the Control condition, the association remained and feeling more suspicious was associated with feeling less accepted the next moment both pre- (b = −.07; 95%CI −.12, −.02, p = 0.004) and post-measurement (b = −.08; 95%CI −.14, −.03, p = 0.003).

No difference was found between MBCT compared to Control with respect to the effect of social acceptance on prospective feelings of paranoia between pre- and post-treatment (b = .04; 95%CI −.06, .14, p = 0.43). Overall, feeling less accepted resulted in feeling more suspicious the next moment (b = −.06; 95%CI −.08, −.03, p<0.001), illustrating the vicious circle between these two variables (see Figure 1 and Figure 2). All results remained significant and similar when controlling for momentary paranoia at t.

## Discussion

This is the first daily life study to show that MBCT changes the way in which individuals with affective dysregulation deal with feelings of insecurity and paranoia in the social world. Our results indicate that MBCT decreases feelings of paranoia in the flow of daily life, while paranoia grew worse in the control group. Moreover, MBCT significantly increased feeling accepted in social situations while controls reported reduced social acceptance from baseline to follow-up. A bi-directional link between feeling paranoid and feeling accepted was apparent, which was attenuated as a result of MBCT, suggesting that MBCT interrupts the vicious circle of feeling paranoid and not feeling accepted (see Figure 1 and Figure 2). This interruption seems to particularly take place between paranoia and social acceptance the next moment, but not between feeling accepted on paranoid feeling the next moment.

Improvements in social interaction (because of their central importance for human beings) may be one of the indirect mechanisms underlying the beneficial effects of mindfulness-based interventions in depression. Feeling paranoid and not accepted during social interaction may become detrimental for wellbeing, leading to increased isolation [9]. Earlier work shows that dispositional and state mindfulness predict self-regulatory behaviour and positive emotional states [36]. Thereby, MBCT may prevent individuals from becoming socially isolated and developing additional symptoms, for example social anhedonia. Moreover, the interpretation of events is at the heart of affective dysregulation, according to influential cognitive theories [37]. MBCT may weaken the impact of paranoia on affective dysregulation by promoting a different relationship to intrusive thoughts (aware yet detached; [38]) as well as self-compassion [39]. By affecting paranoia and its impact on a network of are causally linked symptoms [40], MBCT may be able to prevent a cascade of symptoms that would normally result in a worse prognosis and increased risk for need for care in individuals with affective dysregulation. Therefore, MBCT may be particularly suited for individuals suffering from (sub-clinical) paranoia. In early intervention, circular processes that otherwise would take place and induce shifts upward the psychosis continuum may be prevented. ESM is ideally suited to examine these interactions at the most fundamental “micro-level” expression of psychopathology [41] and to examine directionality of momentary processes and the smallest building blocks that may eventually form part of a mental illness. More research is necessary to investigate whether MBCT also improves feeling accepted during social interaction when feelings of paranoia are more pronounced (e.g., as in individuals in an acute psychotic episode or at ultra-high risk to develop a psychotic disorder).

Only a handful of small RCTs using mindfulness-based therapies in participants with psychotic symptoms exist and it is difficult to compare the current study with earlier work because of differences in sample characteristics (sub-clinical feelings of suspiciousness vs. psychotic patients) and employed methodology (experience sampling vs. retrospective questionnaires). Two small randomized trials showed that ACT significantly reduces distress related to hallucinations [42], [43] as well as re-hospitalization for in-patients with psychosis [18], with effects extending to up to 1 year post-discharge [44]. Three other studies in individuals with a psychotic disorder examined the effect of MBCT [14], [15], [16] compared to waiting list control conditions and found improvements in clinical functioning and mindfulness. Recently, improvements in psychological symptoms, command hallucinations, negative symptoms, depression and crisis contacts were reported after mindfulness-based therapies in psychosis [6], [45], [46]. Our findings extend these findings to real-life paranoia in the context of major mood disorder, and provide a framework for an understanding of the (psychological) mechanisms underlying these positive clinical changes.

Some strengths and limitations of the current RCT should be mentioned. An important observation is that conclusions are limited to subclinical paranoia. One limitation concerns the absence of an active control group. Moreover, we had no measure of MBCT fidelity with respect to both adherence and competence. For some of the ESM variables used in the current study, single item measures were used. ESM requires selectivity in choosing one's items, because participants have to answer them so frequently (in this study, up to 120 times). While construct validity may suffer due to these restrictions, ecological validity and reliability of the estimation are strongly boosted [47]. These particular single-item measures items have been used in several ESM studies in our research group (e.g. [31]) and have demonstrated predictive and ecological validity. The momentary paranoia scores of the current sample with residual mood complaints were elevated compared to the general population, but were below the momentary paranoia scores of (paranoid) psychotic patients.Strengths include the large sample size and sufficient power for the analysis of group by time interaction effects. Examining (subclinical) paranoid ideas in a sample with affective dysregulation has the advantage that patterns underlying psychosis remain relative plastic, so that changes in psychological processes related to paranoia may be investigated more easily. The most important asset of the current study is the use of repeated assessment of paranoia and social acceptance in daily life, enabling fine-grained examination of changes in psychological processes.

## Supporting Information

Checklist S1 **CONSORT Checklist.**
(DOC)

Protocol S1 **Trial Protocol.**
(PDF)
