# Supplementary material for: Putting a Hold on the Downward Spiral of Paranoia in the Social World: A Randomized Controlled Trial of Mindfulness-Based Cognitive Therapy in Individuals with a History of Depression
Source: PLoS One. 2013 Jun 27;8(6):e66747. doi: 10.1371/journal.pone.0066747 (PMC3694971; doi:10.1371/journal.pone.0066747)
Supplement: Protocol S1 — Trial Protocol. (PDF) [file pone.0066747.s002.pdf]

RESEARCH PROTOCOL

(September 2007)

Gene-person-environment interaction in resilience against depression

Protocol ID	gene-person-environment interaction in resilience against depression
Short title	The etiology of resilience against depression
Version	1
Date	7th of September 2007
Coordinating investigator/project leader	M.C Wichers, Dept Psychiatry & neuropsychology, Vijverdal (concorde building)<br>Tel: 043-3688669 Fax: 043-3688689
Principal investigator(s) (in Dutch: hoofdonderzoeker/uitvoerder)	M.C Wichers, Dept Psychiatry & neuropsychology, Vijverdal (concorde building)<br>Tel: 043-3688669 Fax: 043-3688689
Sponsor (in Dutch: verrichter/opdrachtgever)	M.C. Wichers
Independent physician(s)	Dhr. F. van Dael
Laboratory sites <*if applicable*>	Not applicable
Pharmacy <*if applicable*>	Not applicable

PROTOCOL SIGNATURE SHEET

Name	Signature	Date
Sponsor or legal representative:<br>*<please include name and function>*<br><br>For non-commercial research,<br>Head of Department:<br>*<include name and function>*	J.J. Van Os, head of department Psychiatry & Neuropsychology	7th of September 07
Coordinating Investigator/Project leader/Principal Investigator:<br>*<please include name and function>*	M.C. Wichers, post-doc research fellow	7th of September 07

TABLE OF CONTENTS

1. INTRODUCTION AND RATIONALE 9
2. OBJECTIVES 11
3. STUDY DESIGN 12
4. STUDY POPULATION 13
4.1 Population (base) 13
4.2 Inclusion criteria 13
4.3 Exclusion criteria 13
4.4 Sample size calculation 13
5. TREATMENT OF SUBJECTS 14
5.1 Investigational product/treatment 14
5.2 Use of co-intervention (if applicable) 14
5.3 Escape medication (if applicable) 14
6. INVESTIGATIONAL MEDICINAL PRODUCT 14
7. METHODS 15
7.1 Study parameters/endpoints 15
7.1.1 Main study parameter/endpoint 15
7.1.2 Secondary study parameters/endpoints (if applicable) 15
7.1.3 Other study parameters (if applicable) 15
7.2 Randomisation, blinding and treatment allocation 15
7.3 Study procedures 16
7.4 Withdrawal of individual subjects 16
7.4.1 Specific criteria for withdrawal (if applicable) 16
7.5 Replacement of individual subjects after withdrawal 17
7.6 Follow-up of subjects withdrawn from treatment 17
7.7 Premature termination of the study 17
8. SAFETY REPORTING 17
9. STATISTICAL ANALYSIS 17
9.1 Descriptive statistics 17
9.2 Univariate analysis 17
9.3 Multivariate analysis 17
9.4 Interim analysis (if applicable) 17
10. ETHICAL CONSIDERATIONS 18
10.1 Regulation statement 18
10.2 Recruitment and consent 18
10.3 Objection by minors or incapacitated subjects (if applicable) 18
10.4 Benefits and risks assessment, group relatedness 18
10.5 Compensation for injury 19
10.6 Incentives (if applicable) 19
11. ADMINISTRATIVE ASPECTS AND PUBLICATION 20
11.1 Handling and storage of data and documents 20
11.2 Amendments 20

11.3 Annual progress report....20
11.4 End of study report....20
11.5 Public disclosure and publication policy....21
12. REFERENCES....22

LIST OF ABBREVIATIONS AND RELEVANT DEFINITIONS

ABR	ABR form (General Assessment and Registration form) is the application form that is required for submission to the accredited Ethics Committee (ABR = Algemene Beoordeling en Registratie)
AE	Adverse Event
AR	Adverse Reaction
CA	Competent Authority
CCMO	Central Committee on Research Involving Human Subjects
CV	Curriculum Vitae
DSMB	Data Safety Monitoring Board
EU	European Union
EudraCT	European drug regulatory affairs Clinical Trials GCP Good Clinical Practice
IB	Investigator's Brochure
IC	Informed Consent
IMP	Investigational Medicinal Product
IMPD	Investigational Medicinal Product Dossier
METC	Medical research ethics committee (MREC); in Dutch: medisch ethische toetsing commissie (METC)
(S)AE	Serious Adverse Event
SPC	Summary of Product Characteristics (in Dutch: officiële productinfomatie IB1-tekst)
Sponsor	The sponsor is the party that commissions the organisation or performance of the research, for example a pharmaceutical<br>company, academic hospital, scientific organisation or investigator. A party that provides funding for a study but does not commission it is not regarded as the sponsor, but referred to as a subsidising party.
SUSAR	Suspected Unexpected Serious Adverse Reaction
Wbp	Personal Data Protection Act (in Dutch: Wet Bescherming Persoonsgevens)
WMO	Medical Research Involving Human Subjects Act (Wet Medisch-wetenschappelijk Onderzoek met Mensen

SUMMARY

Rationale: The experience of positive emotions reduces daily life stress-sensitivity, an endophenotype for depression, but also attenuates the expression of genetic risk for depression. This research proposal, therefore, will focus on the changeability (plasticity) of the ability to experience positive emotions in response to daily life events (natural rewards), as a first step towards novel (preventive) interventions in depression

Objective:

1) Can the ability to experience reward in daily life be experimentally modified?

2) How does experimental modification of reward impact on depressive symptomatology and can individual variation be traced to genetic variation.

Study design: An intervention study is used in which subjects are randomized to treatment as usual (TAU) or TAU + mindfulness-based cognitive therapy. Experience Sampling Method (ESM) is a structured diary, momentary assessment technique to study subjects in their daily life. Before and after the intervention subjects undergo a six-day period of ESM assessment. Subjects will be globally informed, but remain blind as to how ESM measures relate to testing the hypothesis.

Study population: A sample of 120 depression-remitted adult subjects with residual symptomatology (Ham-17>7) recruited from the community mental health centre in Maastricht (CMHC).

Intervention (if applicable): The experimental group receives 8 weeks of mindfulness training by an experienced trainer in addition to their normal treatment, if any. Sessions are weekly (2,5 hours a session) and subjects receive daily homework exercises. The control group continues their normal treatment, if any.

Main study parameters/endpoints: The increase in reward experience in daily life, whereby reward experience is conceptualised as the effect of small daily life positive events on positive mood state. Since ESM measurements are performed 10 times a day for 6 days, there will be a maximum of 60 measurements within each subject concerning effects of positive events on positive mood state in the flow of daily life.

Nature and extent of the burden and risks associated with participation, benefit and group relatedness: There are no health risks associated with participation. There is a personal benefit for subjects in that they are randomized to receive an extra training that is expected to improve their resilience against depression. Time investment for subjects is 2,5 hours for questionnaires (distributed over three meetings, including the screening). In addition, they participate twice in the ESM protocol (6-day period of filling in dairies concerning daily life events and mood). Before the start each ESM protocol they have to come for a short meeting (15 -30 min) to receive specific instructions related to this

procedure. Of course the subjects (in the experimental condition) also have to participate in the MBCT training and invest time there.

1 INTRODUCTION AND RATIONALE

The bulk of depression research is on negative mood states with little attention to positive emotions. Positive affect (PA) and negative affect (NA) are not two extremes within a unidimensional construct, but relatively independent[1], and there is evidence that the capacity to experience positive emotions may have strong protective effects against depression[2,3]. According to the “broaden and build” theory[4,5], the experience of positive emotions at times of stress reduces the likelihood of developing negative mood outcomes. The reason for this is, first, that positive emotions like joy, interest, contentment and love stimulate exploration of the environment and interaction with others, thus increasing the subjects’ physical and social resources. Second, positive emotions, in contrast to their negative counterparts, increase the attentional focus, which helps people make use of the positive elements, or natural rewards, present in the environment at times of stress, as opposed to narrowing the attentional focus to the negative component of the situation. The process of attentional broadening thus likely prevents people falling into a spiral of negative thinking.

My previous work leading to the proposal

In a twin study (n=279 twin pairs)[6], I identified a depression endophenotype of negative mood states (Y-axis figures below) in response to minor stressors (X-axis figures below) measured in the flow of daily life (this negative mood response to minor stressors is hereafter referred to as “negative affective reactivity”; figure left). In the same twin sample, I tested the hypothesis that experience of positive emotions at the time of the stressor decreases negative affective reactivity. The data confirmed this hypothesis: PA protected subjects from developing negative mood states in response to daily life minor stressors[7] (figure right). In addition, PA at the time of the momentary stress experience also attenuated the association between genetic risk for depression (expressed as co-twin depression status) and negative affective reactivity[7]. The experience of PA may thus be of prime importance in mitigating both symptoms of, and expression of genetic risk for, depression.

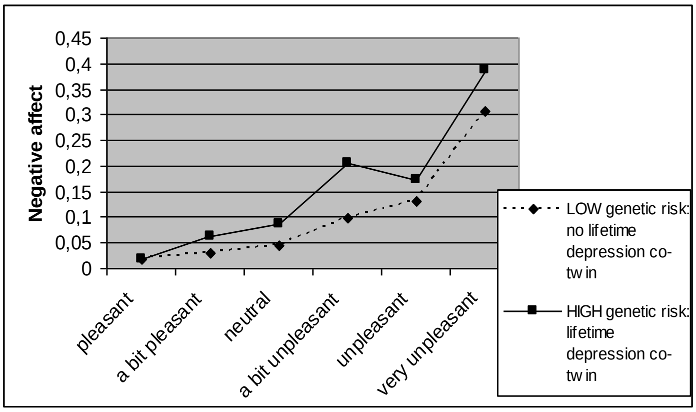

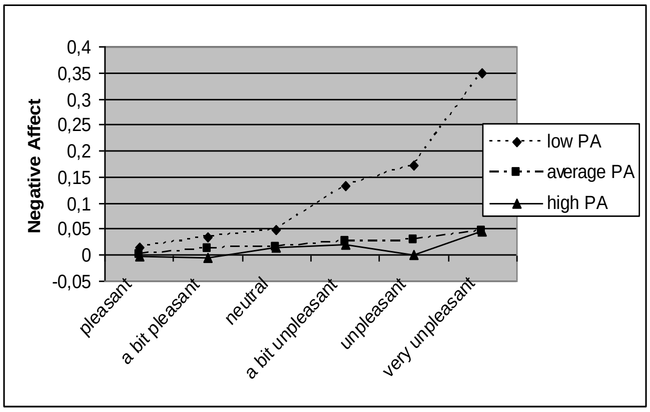

In order to establish possible clinical relevance of these findings, it becomes necessary to i) quantify the extent to which people are able to use natural rewards in the environment in order to generate PA, and ii) establish whether this ability can be learned. To this end I operationalised, using the Experience Sampling Method (ESM; see below), the ability to

experience positive emotions from everyday life situations as the *effect of minor events on positive affect in the flow of daily life*[7] (hereafter: reward experience).

I hypothesize that learning to experience positive emotions from daily events is i) possible and ii) may protect against (risk for) depression, examining i) the degree of plasticity of the ability to experience reward (can it be learned), ii) its impact on residual depression and iii) genetic associations with the brain reward system.

The brain reward system

Depressed subjects have impaired reward function[8] (anhedonia or inability to experience reward[9]). The brain reward system involves structures of the mesocorticolimbic system, with dopamine pathways originating in the ventral tegmental area (VTA) and projecting towards the nucleus accumbens (NAc), limbic and cortical areas[8].
Recent studies[10-14] suggest that manipulations of key proteins involved in the mesolimbic reward (VTA –NAc) circuit of rodents (e.g. dopamine (DA), CREB, kappa opioid receptor, BDNF) produce unique behavioral phenotypes, some of which may be relevant to depression. I will study genetic variation related to these in association with my ESM momentary assessment paradigm of reward mechanisms in the flow of daily life (see below).

2 OBJECTIVES

Primary Objective:
Can the ability to experience positive emotions from everyday life situations assessed with ESM (reward experience), be experimentally modified?
Secondary Objectives:
ii) Will experimentally induced increases in reward experience in daily life reduce depressive symptoms in patients with residual symptomatology?
iii) Are polymorphisms of genes coding for key proteins in the mesolimbic reward system associated with reward experience in daily life and acquisition of this ability?

Depressed subjects with (prevalent and harmful[15,16]) residual symptomatology will be examined. The rationale for this is that examining modification of reward experience in these subjects may additionally shed light on its clinical relevance, i.e. the degree of associated reduction of residual symptoms.

3. STUDY DESIGN

Experience Sampling Method
ESM is a structured diary, momentary assessment technique to study subjects in their daily life. Subjects receive a diary, containing a set of ESM self-assessment forms (rated on 7-point Likert scales) and a digital watch. Ten times a day on six consecutive days, the watch emits a signal at unpredictable moments between 7.30 a.m. and 10.30 p.m. To assess positive affect in responses to environmental situations, reports of current context (activity, persons present, location), rumination, appraisals of the current situation and mood are collected after each "beep". Previously, I operationalised reward experience as the *effect of minor events on positive affect*[7]. ESM studies individuals prospectively and longitudinally, with short time delays between occurrence and assessment of experiences, minimalising biases associated with selective forgetting, attributional style or knowledge about posterior events. I have ample experience with ESM; its validity and feasibility have been extensively documented by our group[17].

Design
Subjects are randomised to two groups. At baseline, all subjects will undergo a six-day period of ESM. Then, one group receives MBCT (8 weekly sessions of two hours including daily homework exercises) by an experienced MBCT therapist at the Community Mental Health Centre in Maastricht (CMHC) in addition to care as usual, and the other group only care as usual, followed by a second six-day ESM assessment. Any additional treatment, if applicable, will be kept constant within subjects. Subjects will be globally informed, but remain blind as to how ESM measures relate to testing the hypothesis.

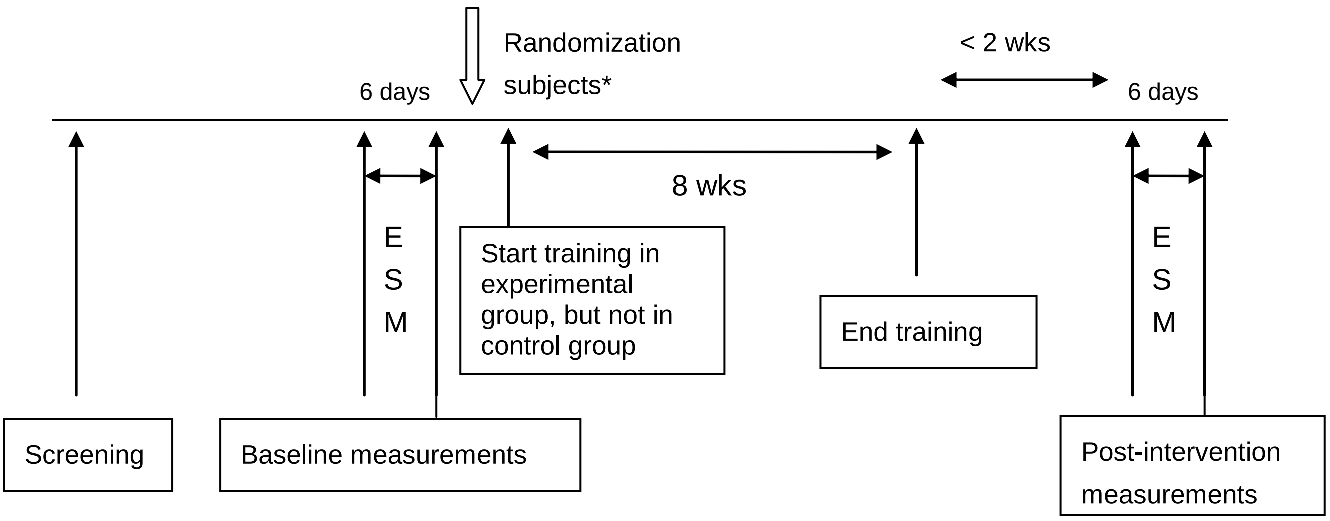

***** Subjects are randomized in two groups. The only difference between these two groups is that the experimental group receives 8 weeks of MBCT training and the control group does not. Subjects of both groups continue with care as usual, if any.

4. STUDY POPULATION

4.1 Population (base)

A sample of 120 depression-remitted subjects with residual symptomatology will be included, recruited from the CMHC in Maastricht, The Netherlands.

4.2 Inclusion criteria:

We want to include adult subjects with:
1. a previous episode of major depressive disorder according to DSM-IV criteria
2. residual depressive symptoms: score>7 on Hamilton Depression Rating Scale (HAM-17), for at least two weeks.

4.3 Exclusion criteria

Fulfilling criteria for major depressive disorder according to DSM-IV criteria at the current moment.

4.4 Sample size calculation

A power simulation shows that for the least powerful analysis (gene-training effect interaction concerning the polymorphism with the most unequally divided genotypes), the power to detect interaction of small-moderate effect size (0.4) is still 80%. As I will use momentary assessment techniques yielding (6x10x2=) 120 different outcomes within each subject, a relatively small sample is needed for sufficient power to examine power-hungry gene-environment interactions

5 TREATMENT OF SUBJECTS

5.1 not applicable

5.2 Investigational product/treatment

Reward modification tool

The tool to modify reward experience, the intervention, is a technique called *Mindfulness-based Cognitive Therapy* (MBCT). During MBCT, people are trained towards increased moment-to-moment awareness of experience, resulting in increased openness or receptiveness[18]. This training is based on meditation techniques to train deployment of attention in a way that subjects learn to focus attention on the present (to be mindful). Thus, during MBCT people train (by doing attention exercises) the ability to focus or detach attention. This will help people to remain open to what is there, to experience it fully without aversion or attachment. In that sense, the training is not like psychotherapy and does not involve talking about life experiences. Although many studies show that MBCT is effective in reducing depressive symptoms and relapse probability[18-20], the mechanism remains unknown. MBCT is also used for treatment of chronic pain or burn-out and in healthy subjects to increase wellbeing. I hypothesise that MBCT is a tool that increases the ability to make use of natural, moment-to-moment rewards in the environment.

5.3 Use of co-intervention (if applicable)

Not applicable

5.4 Escape medication (if applicable)

6 INVESTIGATIONAL MEDICINAL PRODUCT

Not applicable

7. METHODS

7.1 Study parameters/endpoints

7.1.1 Main study parameter/endpoint

-The increase in reward experience in daily life, where reward experience is conceptualized as the effect of small positive daily life events on positive mood.
Since ESM is measures are performed 10 times a day for 6 days, there will be a maximum of 60 measurements within each subject concerning the effects of positive events on positive mood in the flow of daily life.

7.1.2 Secondary study parameters/endpoints (if applicable)

-The association between increase in reward experience and decrease in depressive symptomatology.
- The association between individual variation in reward experience (or increase in reward experience) and genetic variation in polymorphisms related to the brain reward system.

Genetic variation is measured in the collected saliva. Since insights concerning relevant polymorphisms and haplotypes change rapidly, an up-to-date relational (i.e. cross-relating genotype, phenotypic expressions, biochemical pathways) database relevant for the neurosciences is maintained within our department. Genetic analyses thus are always matched against the most recent information. According to current scientific knowledge we would decide to measure polymorphisms in the dopamine receptor 2 gene, the catechol-O-methyl transferase (COMT) gene, human kappa opioid receptor gene and the brain-derived-neurotrophic factor (BDNF) gene.

7.1.3 Other study parameters (if applicable)

Possible moderators of main study parameter:

- number and recency of previous depressive episodes
- age of onset first depressive episode
- life events and childhood trauma
- personality

Possible confounders/mediators:

- amount of rumination
- baseline and change in negative affect

7.2 Randomisation, blinding and treatment allocation

Subjects are randomized to MBCT + treatment as usual (experimental group) or treatment as usual (control group). Randomization takes place after the baseline measurements and first week of ESM. This is because ESM measurements are not

considered valid in case <30% of the responses are valid. Subjects that have not enough valid ESM response are excluded from the study before randomization. Subjects are obviously not blind to their treatment allocation, however, they do remain blind as to how ESM measures relate to testing the hypothesis.

7.3 Study procedures

First, subjects are screened for the participation criteria and fill in a questionnaire concerning demographic variables.
At baseline subjects:
- undergo Psychiatric interviews: SCID-I and the Hamilton Depression Rating Scale (HAM-17).
- fill in some questionnaires concerning depressive symptoms (SCL-90), personality (NEO-PI) rumination (RRS), life events (Interview for Recent Life Events) and early adversity (Childhood Trauma Questionnaire)
- spit some saliva in special tubes as to measure polymorphisms/haplotypes in the DNA that have been associated in previous studies with the brain reward system. (Since insights concerning relevant polymorphisms and haplotypes change rapidly, an up-to-date relational (i.e. cross-relating genotype, phenotypic expressions, biochemical pathways) database relevant for the neurosciences is maintained within our department. Genetic analyses thus are always matched against the most recent information).
- participate in the 6-day ESM period as described under 'study design'. Subjects will be briefed and de-briefed before and after the ESM period, respectively.

Subjects randomized to the experimental group will participate in an 8-week-period of MBCT, including weekly sessions of 2,5 hours in which the training takes place and daily homework exercises. They also fill in to what degree they complied with the homework exercises every day (in minutes).

After the intervention, subjects from both groups (experimental and control) again undergo the psychiatric interviews and the questionnaires.
In addition, they participate in another 6-day ESM period and are again briefed and de-briefed with respect to this procedure. The ESM debriefing includes the collection of the diaries and discussion concerning unclarities or missing values.

7.4 Withdrawal of individual subjects

Subjects can leave the study at any time for any reason if they wish to do so without any consequences. The investigator can decide to withdraw a subject from the study for urgent medical reasons.

7.4.1 Specific criteria for withdrawal (if applicable)

Not applicable

7.5 Replacement of individual subjects after withdrawal

In case subjects withdraw before randomization, they will be replaced. After randomization they will not be replaced. The power calculation takes into account a certain amount of drop-out from the study.

7.6 Follow-up of subjects withdrawn from treatment

Subjects that withdraw from treatment will be asked the reason of withdrawal. In addition, it will be examined whether these subjects differ in any characteristics from those that do not withdraw.

7.7 Premature termination of the study

Not applicable

< Please describe the criteria for terminating the study prematurely and the procedures in case the study will be terminated prematurely.>

8 SAFETY REPORTING

Not applicable

9 STATISTICAL ANALYSIS

9.1 Descriptive statistics

Frequency tables and chi-square tests are used for categorical data and means and standard deviations for continuous variables.

9.2 Univariate analysis

Analyses are corrected for possible confounders, thus only multivariate analyses will be used.

9.3 Multivariate analysis

ESM data have a hierarchical structure. Thus, multiple observations (level 1) were clustered within subjects (level 2). Multilevel analysis takes the variability associated with each level of nesting into account [21]. The XTMIXED command in STATA 9.1 (Statacorp, College Station, Tex) was used to perform multilevel linear regression analyses.

9.4 Interim analysis (if applicable)

not applicable

10 ETHICAL CONSIDERATIONS

10.1 Regulation statement

The study will be conducted according to the principles of the Declaration of Helsinki and in accordance with the Medical Research Involving Human Subjects Act (WMO*).*

10.2 Recruitment and consent

Recruitment will take place at the community mental health centre in Maastricht. Subjects will be made aware of the possibility to participate in this study by their supervising care givers. In case subjects show interest they will be invited by the investigator of this study who will provide them with all information about the study and risks, benefits and rights of participants. Subjects have a week to decide whether or not they want to participate. In case a subject does not want to participate, but changes his/her mind later, he or she can still participate.

10.3 Objection by minors or incapacitated subjects (if applicable)

Not applicable. Only adults (>18 years) and capacitated individuals will be included in the study.

10.4 Benefits and risks assessment, group relatedness

There are no health risks associated with the research.

There is a personal benefit for subjects in that they are offered to receive an extra training that is expected to improve their resilience against depression. There is scientific evidence for the notion that MBCT training has positive effects on mental health and decreases strongly the relapse rate for depression (with around 20-30%, leaving only half of the normal relapse rate) in subjects in remission of depression[18,20,22].

There is a scientific benefit in that it leads to greater insight into the biological and psychological factors related to resilience, which will be important for prevention of depressive disorder in the population.

Burden for the patients is some time investment. After the screening subjects have to come twice for a meeting in which they are interviewed concerning depressive symptoms and fill in some questionnaires (2 x 1 hour). At the baseline meeting subjects have to spit some saliva in a tube. This will take only a minute. They participate twice in the ESM protocol (6-day period of filling in dairies concerning daily life events and mood). This procedure will costs approximately 4,5 hours in total for the two periods. Before the start of each ESM protocol they have to come for a short meeting (15 -30 min) to receive specific instructions related to this procedure.

10.5 Compensation for injury

There are no health risks related to this study. Dispensation from the statutory obligation to provide insurance is requested.

10.6 Incentives (if applicable)

Subjects will receive free MBCT training. Subjects that are randomized to enter the control group are offered an MBCT course that they can follow later, after the study. In addition, they receive some financial compensation in relation to the amount of time they invested in the study, with a maximum of 50 euro. In addition, subjects are fully compensated for any travel costs related to the study.

11 ADMINISTRATIVE ASPECTS AND PUBLICATION

11.1 Handling and storage of data and documents

Data are handled confidentially and if possible anonymously. The data are coded using a number indicating the order of entry. Only the main investigator (M.C. Wichers) has permission to access the source data. Where it is necessary to be able to trace data to an individual subject, a subject identification code list is used to link the data to the subject. The key to the code is safeguarded by the investigator. Collected material is coded and will be stored with the purpose of analysis. After material is analysed it will be destroyed. The handling of personal data complies with the Dutch Personal Data Protection Act (in Dutch: De Wet Bescherming Persoonsgegevens, Wbp).

11.2 Amendments

<*The following text is applicable for studies without an investigational medicinal product*.>
Amendments are changes made to the research after a favourable opinion by the accredited METC has been given. All amendments will be notified to the METC that gave a favourable opinion.

All substantial amendments will be notified to the METC and to the competent authority.

Non-substantial amendments will not be notified to the accredited METC and the competent authority, but will be recorded and filed by the sponsor.

11.3 Annual progress report

The sponsor/investigator will submit a summary of the progress of the trial to the accredited METC once a year. Information will be provided on the date of inclusion of the first subject, numbers of subjects included and numbers of subjects that have completed the trial, serious adverse events/ serious adverse reactions, other problems, and amendments.

11.4 End of study report

<*The following text is applicable for studies without an investigational medicinal product*.>

The investigator will notify the accredited METC of the end of the study within a period of 8 weeks. The end of the study is defined as the last patient's last visit.

In case the study is ended prematurely, the investigator will notify the accredited METC, including the reasons for the premature termination.

Within one year after the end of the study, the investigator/sponsor will submit a final study report with the results of the study, including any publications/abstracts of the study, to the accredited METC.

11.5 Public disclosure and publication policy

Results of this study will be published in peer-reviewed international scientific journals. Both positive and negative results will be published. There are no restrictions in the public disclosure of scientific findings resulting from this study.

12 REFERENCES

1. Bradburn N. The structure of psychological well-being. Chicago: Aldine, 1969.
2. Strand EB, Zautra AJ, Thoresen M, Odegard S, Uhlig T, Finset A. Positive affect as a factor of resilience in the pain-negative affect relationship in patients with rheumatoid arthritis. *J Psychosom Res* 2006;**60**(5)**:**477-84.
3. Tugade MM, Fredrickson BL. Resilient individuals use positive emotions to bounce back from negative emotional experiences. *J Pers Soc Psychol* 2004;**86**(2)**:**320-33.
4. Fredrickson BL. What good are positive emotions. *Review of General Psychology* 1998;**2**(3)**:**300-319.
5. Fredrickson BL. The role of positive emotions in positive psychology. The broaden-and-build theory of positive emotions. *Am Psychol* 2001;**56**(3)**:**218-26.
6. Wichers MC, Myin-Germeys I, Jacobs N, et al. Evidence that Genetic Risk for Depression is Expressed as Mood Bias to Negative Emotions in the Flow of Daily Life: A Momentary Assessment Twin Study. *British Journal of Psychiatry* in press.
7. Wichers MC, Myin-Germeys I, Jacobs N, et al. Evidence that moment-to-moment variation in positive emotions buffer genetic risk for depression: A momentary assessment twin study. *Acta Psychiatr Scand* in press.
8. Naranjo CA, Tremblay LK, Busto UE. The role of the brain reward system in depression. *Prog Neuropsychopharmacol Biol Psychiatry* 2001;**25**(4)**:**781-823.
9. APA. Mood disorders. DSM-IV Diagnostic and Statistical Manual of Mental Disorders
4th ed. Washington, DC, 1994: 161-198.
10. Nestler EJ, Carlezon WA, Jr. The mesolimbic dopamine reward circuit in depression. *Biol Psychiatry* 2006;**59**(12)**:**1151-9.
11. Barrot M, Olivier JD, Perrotti LI, et al. CREB activity in the nucleus accumbens shell controls gating of behavioral responses to emotional stimuli. *Proc Natl Acad Sci U S A* 2002;**99**(17)**:**11435-40.
12. Carlezon WA, Jr., Thome J, Olson VG, et al. Regulation of cocaine reward by CREB. *Science* 1998;**282**(5397)**:**2272-5.
13. Mague SD, Pliakas AM, Todtenkopf MS, et al. Antidepressant-like effects of kappa-opioid receptor antagonists in the forced swim test in rats. *J Pharmacol Exp Ther* 2003;**305**(1)**:**323-30.

14. Eisch AJ, Bolanos CA, de Wit J, et al. Brain-derived neurotrophic factor in the ventral midbrain-nucleus accumbens pathway: a role in depression. *Biol Psychiatry* 2003;**54**(10)**:**994-1005.
15. Judd LL, Akiskal HS, Maser JD, et al. Major depressive disorder: a prospective study of residual subthreshold depressive symptoms as predictor of rapid relapse. *J Affect Disord* 1998;**50**(2-3)**:**97-108.
16. Kennedy N, Paykel ES. Residual symptoms at remission from depression: impact on long-term outcome. *J Affect Disord* 2004;**80**(2-3)**:**135-44.
17. Jacobs N, Nicolson NA, Derom C, Delespaul P, van Os J, Myin-Germeys I. Electronic monitoring of salivary cortisol sampling compliance in daily life. *Life Sci* 2005;**76**(21)**:**2431-43.
18. Grossman P, Niemann L, Schmidt S, Walach H. Mindfulness-based stress reduction and health benefits. A meta-analysis. *J Psychosom Res* 2004;**57**(1)**:**35-43.
19. Ma SH, Teasdale JD. Mindfulness-based cognitive therapy for depression: replication and exploration of differential relapse prevention effects. *J Consult Clin Psychol* 2004;**72**(1)**:**31-40.
20. Teasdale JD, Segal ZV, Williams JM, Ridgeway VA, Soulsby JM, Lau MA. Prevention of relapse/recurrence in major depression by mindfulness-based cognitive therapy. *J Consult Clin Psychol* 2000;**68**(4)**:**615-23.
21. Snijders T, Bosker R. Multilevel analysis: an introduction to basis and advanced multilevel modeling. London: SAGE publications Ltd, 1999.
22. Reibel DK, Greeson JM, Brainard GC, Rosenzweig S. Mindfulness-based stress reduction and health-related quality of life in a heterogeneous patient population. *Gen Hosp Psychiatry* 2001;**23**(4)**:**183-92.
